# Nutritionally Improved Traditional Recipes and Fortified Infant Flours to Increase the Nutritional and Energy Intake in 6–11-Month-Old Infants in Rural Niger: A Randomized Controlled Trial

**DOI:** 10.3390/nu18132117

**Published:** 2026-06-29

**Authors:** Faustine Rio-Puygrenier, Christèle Icard-Vernière, Nafiou Maman Ilia Aminou, Mélanie Antoine, Moussa Hainikoye, Haoua Seini Sabo, Sonia Fortin, Claire Mouquet-Rivier

**Affiliations:** 1 IRD (French National Institute for Sustainable Development), BP 64501-911, Av. Agropolis, 34394 Montpellier, France; christele.verniere@ird.fr (C.I.-V.); melanie.antoine@supagro.fr (M.A.); sonia.fortin@ird.fr (S.F.); 2 QualiSud, Univ Montpellier, Avignon Université, CIRAD, Institut Agro, IRD, Université de la Réunion, 34394 Montpellier, France; 3 Faculty of Science and Technology, Abdou Moumouni University, Niamey, Niger; aminouinafiou@yahoo.com (N.M.I.A.); haoua.seini@cermes.org (H.S.S.); 4 Alimentation et Economie Rurale, GRET, Niamey BP 10896, Niger; hainikoye.niger@gret.org

**Keywords:** responsive feeding, fortified complementary food, anemia, micronutrient, infant

## Abstract

Back-ground: In 2022, in Niger, undernutrition was highly prevalent in 6–23-month-old infants and their diet was poorly diversified. Methods: This cluster randomized controlled trial was conducted in the Zinder region of Niger to monitor food and nutritional intakes from two food solutions, fortified infant flours (FIF) and ten nutritionally improved traditional recipes (NITR), in breastfed 6–11-month-old infants divided into four groups: control, responsive feeding (RF) awareness raising, RF + FIF, and RF + NITR. Data were collected at T0 (*n* = 322 infants) and 3 months later (T3, *n* = 300 infants). Results: At T0, 29% and 52% of infants had stunting and anemia, respectively, and 24% of them achieved minimum dietary diversity (MDD) in all groups. At T3, the MDD rates significantly increased, particularly in the RF + FIF and RF + NITR groups (71% and 81%, respectively). Food intake remained low in all groups, below the gastric capacity of children. Nevertheless, at T3, food intake was significantly higher in the RF + NITR group than in the other groups (*p* = 0.0209). Although porridges made with FIF were consumed in smaller quantities, thanks to their high energy density, the mean energy intake was higher in the RF + FIF group than in the control and RF groups. The energy intake of the RF + NITR group was even higher. This can be attributed to the fact that NITR-based meals were more varied, and colorful and offered different tastes and textures, thus appearing more appetizing and stimulating. Conclusions: A strategy that combines FIF and NITR appears relevant for improving nutritional intake in these contexts.

## 1. Introduction

Undernutrition and micronutrient deficiencies are still widespread and concern many young children in low- and middle-income countries. In the 2022 Standardized Monitoring and Assessment of Relief and Transitions (SMART) national survey, in Niger, the prevalence rates of acute malnutrition, stunting and anemia in 6–23-month-old infants were 17.6%, 43.1% and 77.7%, respectively. These rates exceed the thresholds considered very high by the World Health Organization (WHO) [1,2]. Moreover, only 8.7% of 6–23-month-old infants met the minimum dietary diversity (MDD) recommendations [3]. These nutritional problems have many different causes [4] and require improving access to nutritionally rich foods [5]. Young children in Niger have monotonous diets, consisting mainly of breast milk and cereals, and occasionally other food groups [6].

Food fortification and diversification are among the main strategies to improve the energy and nutritional intakes of young children [7]. In Niger, large-scale fortification involves iodized salt, vegetable oils fortified with vitamin A, and wheat flour fortified with iron and folic acid. However, this ultimately has little impact on young children due to their low intake. Fortified infant flours (FIF) are the most commonly used food vehicle for targeted fortification in 6–23-month-old infants [8].

Among the diet diversification strategies, traditional household recipes can be improved by using local ingredients (e.g., pulses, nuts and seeds, flesh foods, dairy products, eggs, fruits and vegetables rich in vitamin A, and other fruits and vegetables) to increase macro- and micro-nutrient intakes. Several studies evaluated the nutritional potential of incorporating legumes and moringa into recipes [9], adding sauces made from leafy vegetables rich in iron and vitamin A [10], or modifying local diets to improve vitamin A, iron and protein intakes [11]. However, their effectiveness on the nutritional status remains limited, and some interventions did not improve iron absorption [12]. Indeed, these studies often focus on the benefits of a specific food or food group, whereas nutritional problems are generally caused by multiple deficiencies.

Moreover, poor appetite is very common in young children in contexts with high prevalence of stunting [13]. Infants and young children often consume small amounts of food during a meal [14,15,16], much smaller than their expected gastric capacity (30 g/kg of body weight (BW)/meal) [17]. Even a nutritionally rich meal must be consumed in sufficient quantities to meet the high nutritional requirements of young children. The WHO recommends adopting responsive feeding (RF) [18], a feeding style that encourages children to eat by promoting positive interactions between the person feeding the child and the child. RF practices have been associated with beneficial effects in <5-year-old children in low- and middle-income countries [16,19] and in young children in Burkina Faso [13].

Therefore, we hypothesized that implementing integrated strategies, including a Water, Sanitation and Hygiene (WASH) package [20], promoting RF practices and the daily consumption of a nutritionally balanced complementary food could contribute more effectively to meet the nutritional and energy requirements of young children, and to reduce the risk of anemia. To test this hypothesis, we carried out a randomized controlled trial (RCT) to compare two food solutions (FIFs and improved home recipes), in addition to breast milk and RF awareness-raising activities, in 322 infants aged 6–11 months in a rural region of Niger. We evaluated the effects of these food solutions on dietary diversity and food and energy intakes and also assessed their energy and nutritional contributions.

## 2. Materials and Methods

### 2.1. Participants, Location and Study Design

The study was conducted in the commune of Dogo, in the Zinder region of eastern Niger between December 2021 (T0) and March 2022 (T3). In this area, the prevalence of stunting (51.5%) and MDD (5.9%) in 6–23-month-old infants was among the highest and lowest in the country, respectively [2].

For this cluster RCT, infants enrolled at the age of 6–7 months were divided in four groups: (i) **control** group (only WASH package); (ii) **RF** group (WASH package and RF promotion); (iii) **RF + FIF** group (WASH package, RF promotion and FIF supply); (iv) **RF + NITR** group (WASH package, RF promotion and food ingredients supply for NITR preparation).

### 2.2. Sample Size and Randomization

Villages were used as randomization units to limit product sharing or inter-group contamination. The forty villages of the Dogo commune were divided into four blocks of similar size, considering the population size and the distance between the village and its Integrated Health Center (IHC). Each block of ten villages was randomly assigned to one of the four intervention groups by means of a draw conducted in the presence of the village chiefs.

The sample size was calculated to detect a meaningful difference in food intake (from 5.0 to 7.5 ± 4 g/kg BW/meal) in 6–23-month-old infants, with a power (1 − β) of 90%, a type I error (α) of 5%, loss to follow-up of 15% and design effect of 2. The required number of children was estimated at 104 per group, for a total of 416 children to be recruited.

### 2.3. Enrolment and Participation

A home-based census was conducted in all selected villages to identify eligible infants, followed by a pre-enrolment visit during which parents were given an appointment at the closest IHC. The 6–7-month-old infants of those who agreed to participate in the study were enrolled in November 2021. To be included in the study, infants had to be breastfed. Exclusion criteria were weight-for-length Z-score < −3 (severe acute malnutrition) and/or presence of bilateral edema, hemoglobin concentration < 7 g/dL (severe anemia), or health problems that could interfere with the study results.

### 2.4. Ethical Consideration

The RCT protocol was approved by the National Health Research Ethics Committee (CNERS) of the Republic of Niger, No. 061/2021/CNERS on 22 October 2021. An information sheet outlining the study objectives, procedures, risks and benefits in a local language was provided and read to the parents, reminding them that their participation was voluntary and could be withdrawn at any time. The parents’ informed consent and the household head’s agreement were obtained by signature. The study protocol was retrospectively registered in the PanAfrican clinical trials registry under the number PACTR202507635630572 (https://pactr.samrc.ac.za/) on 28 July 2025.

### 2.5. Interventions

#### 2.5.1. WASH

Awareness-raising activities on five key hygiene messages were organized at T0 and T3 for all groups (Supplementary Table S1). Families also received a mat to sit their children on for meals and an insecticide-treated mosquito net at T0, and pieces of soap every month.

#### 2.5.2. RF

At T0, the mothers of children in the RF, RF + FIF and RF + NITR groups received bowls graduated according to the portion sizes appropriate for the 6–8 months, 9–11 months and 12–13 months age groups. They were made aware of five key messages to promote RF during group workshops (~10 mothers per workshop), two at T0 and one at T3, and through a home visit by an RF-trained facilitator at T3. Messages were adapted from materials developed for a previous study in Madagascar [16], with changes between T0 and T3 to better meet the needs and developmental stage of 6–8-month-old and 9–11-month-old infants (Supplementary Table S2) and some adaptations to the Niger context.

#### 2.5.3. FIF

Every two weeks, the RF + FIF group received sachets of Misola™ or Garin Yaara™ FIF to prepare two meals of porridge per day (i.e., 70 g of FIF daily). Workshops on how to prepare the fortified porridge were organized. The formulation of the micronutrient premixes (13 vitamins and 12 minerals) was revised beforehand to meet the international recommendations [21]. The proportions of ingredients present in the two FIFs and the nutritional values are in Supplementary Table S3a,b.

#### 2.5.4. NITR

Ten recipes were selected from the National Book of Infant Recipes developed by the Niger Ministry of Public Health in 2019, based on the ingredients available in the study area. This book offers balanced recipes that provide 10–15% of energy from protein, 30–35% from fat and the remaining from carbohydrates. Recipes were adapted to the young children’s requirements by increasing the proportion of nutrient-rich ingredients. The portion sizes for each recipe were calculated to provide 120 kcal for 6–8-month-old infants and 180 kcal for 9–11-month-old infants (i.e., ~60% of the recommended intake from complementary foods at those ages) [18]. Eight recipes were porridges based on millet or maize, one was the traditional millet dumplings enriched with oil, and one was a cowpea purée. Porridges were enriched with one or more ingredients of nutritional interest such as groundnut powder or paste, baobab pulp, cowpea, fish powder, egg yolk, powdered milk or moringa leaf powder.

Every fortnight, the ingredients for home-cooked meals, with the exception of millet and maize, were made available in quantities sufficient for the preparation of two meals per day at the village chiefs’ homes where mothers could collect them. Recipe cards (Supplementary Figure S1) with instructions for preparing a meal were provided, and demonstrations of how to prepare the meals were given by the project leaders.

### 2.6. Measurements

#### 2.6.1. Household Characteristics and Feeding Practices

At T0, a questionnaire was administered to the child’s primary caregiver to collect socioeconomic information about the household. Using multiple correspondence analysis (MCA) on 17 variables related to household housing conditions and asset ownership (Supplementary Table S4a,b), a socioeconomic status score was derived from the first dimension of the MCA [22]. This score was treated as a continuous variable and rescaled from 0 to 100 for interpretability. It was then divided into tertiles using empirical cut-offs based on the 33.3rd and 66.7th percentiles of its distribution, resulting in three equally sized socioeconomic groups ranging from the least affluent (tertile 1) to the most affluent (tertile 3).

Data on breastfeeding and feeding practices were collected at T0 and T3. Morbidity was assessed at T3 using a short questionnaire.

#### 2.6.2. Anthropometry and Hemoglobin Concentrations

At T0 and T3, the infants’ weight and length were measured using the standardized methods recommended by WHO [23] and hemoglobin concentration was assessed using a Hemocue 301 device at the local IHC. The biological mother’s weight and height were measured at T0.

Anthropometric data were processed using the Emergency Nutrition Assessment software (latest version available at the time of analysis) for SMART to calculate the WHZ and HAZ z-scores and determine the prevalence of wasting and stunting, respectively. Anemia was determined, based on the WHO hemoglobin thresholds for 6–11-month-old infants (10.5 g/dL) [24]. The mothers’ body mass index was calculated to determine the prevalence of underweight, based on the WHO thresholds [25].

#### 2.6.3. Food Consumption Data

Two visits at each timepoint were made to the infants’ homes, after making an appointment with the caregiver. To increase robustness, only children with two valid meal observations at a given timepoint were included in the corresponding analyses; children with data from only one visit were excluded from these analyses. And because no significant differences were observed between the two visits at a given timepoint, food intake measurements were averaged to obtain a single value per child and per timepoint.

##### Qualitative 24 h Recall

Dietary diversity based on a qualitative 24 h recall was determined at T0 and T3. Mothers were asked to describe all foods eaten by their child on the day before, following the method recommended by WHO and UNICEF (2021) [3]. Then, dietary diversity scores (DDSs) were calculated considering eight food groups including breastmilk. As recommended by WHO and UNICEF (2021) [3], FIF consumption was scored 1 for the cereal, root and tuber group. However, a DDS variant was created, called FIF-DDS, that took into account the consumption of pulses, nuts and seeds (PNS group). Indeed, the two FIFs contained legumes: soybean and groundnut (Misola) or soybean, cowpea and groundnut (Garin Yaara). The total amount of legumes ingested from FIF-based porridge was calculated (11.4 to 20.9 g) and as it was >10 g, the PNS group was included in the FIF-DDS calculation.

##### Observation of Food Preparation

The investigators recorded all information on the preparation of the meal consumed by the infants (NITRs, FIF porridge, or traditional recipes). The used ingredients were listed and weighed using a Soehnle™ kitchen scale (range 5 kg, accuracy 1 g; Soehnle, Backnang, Germany); methods of preparation and cooking, utensils used, and quantities of food prepared were observed. These observations mainly concerned the RF + FIF and RF + NITR groups because traditional dishes were often prepared in advance in the other groups. For each recipe, the ‘mean observed recipe’ was computed by averaging the amount of each ingredient over all observations of that recipe.

##### Dish Sampling

When possible, samples of the prepared dish were collected and their dry matter (DM) content was measured by weighing after drying in an oven at 105 °C until constant weight.

##### Food Intake Measurements and Calculation of the Energy and Nutrient Intakes

The quantities of food consumed by the infants (i.e., food intake) were measured by weighing the bowl holding the food before and after consumption using a Soehnle™ kitchen scale. The food spilled during consumption was collected using a clean napkin and weighed. The start and end times of consumption were recorded to calculate the meal duration. Mothers were asked about the child’s health and appetite at the time of consumption and about any breastfeeding or food/drink consumed in the hour preceding the meal. The reasons for ending the meal were also recorded.

For each recipe, including FIF porridges, a mean recipe was constructed using the average quantities of each ingredient across preparations. Extreme values in ingredient quantities were identified during data cleaning and excluded to avoid undue influence on the mean recipe composition.

The energy and nutritional values of the mean observed recipes were calculated using the nutritional values of all ingredients taken from the West African Food Composition Table (WAFCT) [26] and taking into account retention factors [27]. The iron content of raw millet was set at 4.51 mg/100 g DM [28] because the value in the WAFCT is very high and not representative of the average iron content in pearl millet.

The measured food intakes were converted into energy intakes based on the recipe energy value. As FIFs and NITR ingredients were provided for two meals, the energy intake was multiplied by two to estimate the contribution to the energy requirements (i.e., 200 kcal/day for 6–8-month-old infants and 300 kcal/day for 9–11-month-old infants) [29]. The contribution of macro- and micro-nutrient intakes to requirements was calculated based on the recommended nutrient intake (RNI) from complementary foods (i.e., the total requirement minus the intake from breastmilk) [17,21,30,31,32].

### 2.7. Data Collection and Entry

Investigators used questionnaires to collect data on the household socio-economic status and food practices, on meal observations and intake measurements. Four women facilitators were in charge of RF awareness-raising activities and cooking demonstrations for NITR. IHC staff carried out the anthropometric measurements and Hemocue tests.

Data were collected on touchscreen tablets via a controlled entry mask, interfaced with the application KoboCollect. All data were sent to the server every evening. When data collection at a given timepoint was completed, data were retrieved from the server for analysis.

### 2.8. Statistical Analysis

Analysis was by a modified intention-to-treat (mITT) or available case analysis for this specific outcome (i.e., all infants were considered according to their initial inclusion group).

This article reports the primary outcomes (i.e., complementary food intakes, in g/meal, and hemoglobin concentrations) and some secondary outcomes (dietary diversity and DM content of complementary foods).

All statistical analyses were performed using R version 4.4.0 (12 January 2025).

Qualitative variables were expressed as percentages. Quantitative variables were first expressed as means and standard deviations (SDs) for the sample characteristics, then, after adjustments, as least squares means (LSs) and standard errors (SEs). Associations were first tested at each timepoint using mixed linear models with intake or anthropometric status as the explanatory variable and the intervention group as a fixed effect. Age, sex, and socioeconomic status terciles were added as fixed adjustment factors, and village and visit as random effects. The intervention impact was evaluated using the same mixed linear models, to which timepoint and its interaction with the group were added as fixed effects. The first type error level was set at 0.05.

The data described in the manuscript and code book are available upon request.

## 3. Results

**Figure 1:**
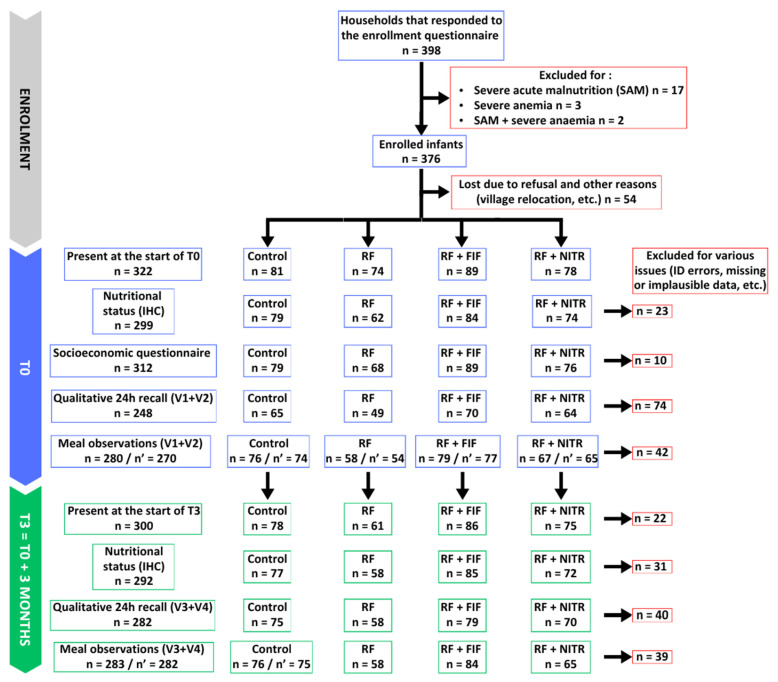
Study population flowchart. IHC = Integrated Health Center. Nutritional status = anthropometric measurements and hemoglobin concentration, V1 + V2 = infants present at both home visits at T0, V3 + V4 = infants present at both home visits at T3, n’ = sample size after excluding observations of meals with a food intake < 10 g.

Of the 398 eligible participants, the caregivers of 376 children gave their written informed consent and met the inclusion criteria (Figure 1). As 54 children and their caregivers finally refused the first food intake measurement at home or moved from their village, the final sample included 322 children.

### 3.1. Sample Characteristics and Nutritional Status

At T0, the infants’ and mothers’ characteristics were similar in the four groups (Table 1). All infants’ caregivers were their biological mothers (mean age: 25.2 ± 6.3 years). On average, they had four living children younger than 18 years; 66% of them had no formal education, 23% were underweight, and only 5% were overweight or obese.

The only difference concerned the socioeconomic status (*p* < 0.001). The least affluent tercile was more represented in the control group, while the most affluent tercile was more represented in the RF + NITR group. However, these differences remained limited because the variables related to housing conditions and possessions were similar among groups (Supplementary Table S4a,b).

In infants, the prevalence of anemia (52%), stunting (29%) and moderate wasting (13%) was high at T0, without significant differences among groups. At T3, the prevalence of stunting increased in all groups, with no significant differences between groups (Supplementary Table S5). Anemia also increased in all groups, but its prevalence was significantly lower (*p* = 0.0246) in the RF + FIF (54%) than in the RF + NITR group (78%).

### 3.2. Dietary Diversity

#### 3.2.1. Dietary Diversity Score

**Figure 2:**
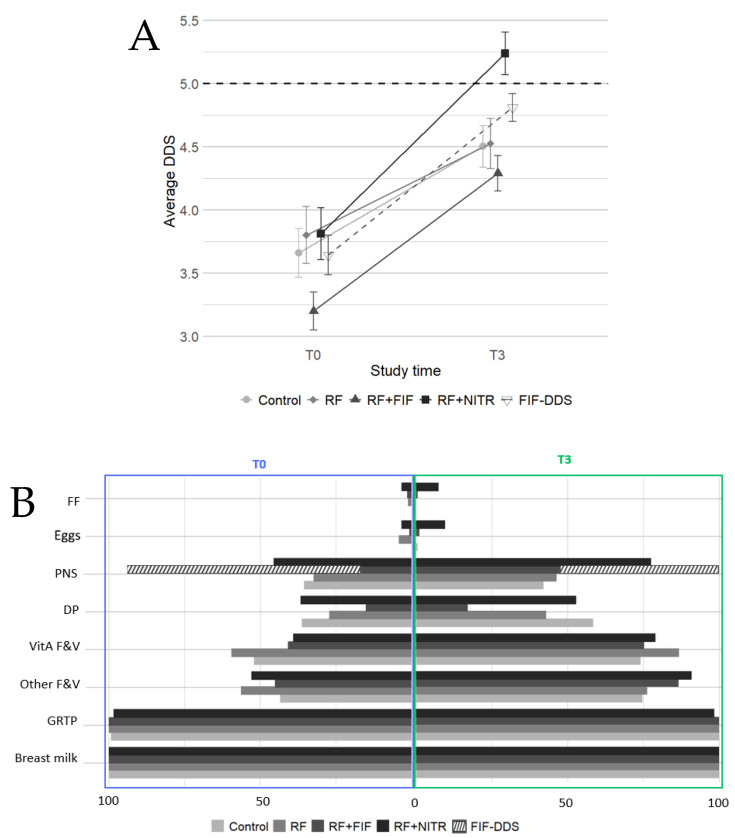
Dietary diversity and food groups consumed. (**A**) Changes in dietary diversity score (DDS) between T0 and T3. The dotted black line corresponds to the minimum dietary diversity threshold (i.e., 5 of 8 food groups consumed in the last 24 h) (WHO & UNICEF, 2021) [3]. (**B**) Food groups consumed by infants (%) at T0 (blue frame) and at T3 (green frame). RF = responsive feeding, FIF = fortified infant flour, NITR = nutritionally improved traditional recipes, FIF-DDS = variant of the DDS recommended by WHO & UNICEF (2021) [3] that takes into account the consumption of the pulses, nuts and seeds (PNS) group when FIF including ingredients from this group are consumed. FF = flesh foods, PNS = pulses, nuts and seeds, DP = dairy products, Other F&V = other fruits and vegetables, VitA-F&V = vitamin A-rich fruits and vegetables, GRTP = grains, roots, tubers and plantains. At T0: Control *n* = 65; RF *n* = 49; RF + FIF *n* = 70; RF + NITR *n* = 64. At T3: Control *n* = 75; RF *n* = 58; RF + FIF *n* = 79; RF + NITR *n* = 70.

Based on the 24 h-recall data, children had a median of four food occasions per day. The DDS increased in all groups between T0 and T3 (Figure 2A) because the children’s age (6–11 months) corresponded to the start of dietary diversification. However, at T0, the mean DDS was <5 and associated with low MDD prevalence (24% for all groups). At T3, the mean DDS was >5 only in the RF + NITR group due to the recipe enrichment with ingredients from various food groups (MDD prevalence of 81%). The mean DDS was significantly lower in the RF + FIF group than in the other groups when calculated according to the international recommendations [3]. However, when legumes (present in significant quantities in the FIFs) were taken into account (FIF-DDS in Figure 2A), the DDS increased and the MDD increased from 44% to 71% in this group and was higher than in the control and RF groups. Indeed, FIF consumption led to a substantial increase in PNS group consumption (Figure 2B).

#### 3.2.2. Food Groups Consumed

All infants were breastfed at both timepoints (Figure 2B).

The grains, roots, tubers and plantains group, which constituted the basis of the infants’ diets from the start of diversification, was consumed by almost all infants at both timepoints, particularly millet.

Consumption of fruits and vegetables, rich in vitamin A or other fruits and vegetables, increased by 50% in all groups, reaching ~80% at T3.

Consumption of dairy products and PNS was very low (~25% of infants at T0 and even ~12% in the RF + FIF group). At T3, their consumption increased to nearly 50% in most groups, except in the RF + FIF group where it remained stable. For the PNS group, consumption was higher (80%) in the RF + NITR group because several NITRs included ingredients from the PNS group. As ingredients from the PNS group were present in significant amounts in the FIFs, they were consumed by 100% of infants in the RF + FIF group.

Egg and flesh foods (i.e., meat and fish) consumption was marginal, although it increased slightly in the RF + NITR group at T3 (10%) because some recipes included these ingredients.

### 3.3. Food Consumption Patterns During the Meal Observation (Table 2)

Overall, meal durations were short, but their duration became significantly different at T3 (*p* < 0.0001). Meal duration was longest (>8 min) in the RF + FIF and RF + NITR groups, followed by the RF group (7.0 min), itself higher than in the control group (5.3 min; *p* = 0.0435).

**Table 2:** Food and energy intakes and consumption patterns during the meal observation at T0 and T3.

	**T0**											**T3**											
	**Control**		**RF**		**RF + FIF**		**RF + NITR**		**Total**		**p_group_ ^a^**	**Control**		**RF**		**RF + FIF**		**RF + NITR**		**Total**		**p_group_ ^a^**	p_group*time_ ^b^
	***n***	**%**	***n***	**%**	***n***	**%**	***n***	**%**	***n***	**%**		***n***	**%**	***n***	**%**	***n***	**%**	***n***	**%**	***n***	**%**		
	**LS Mean ± SE**		**LS Mean ± SE**		**LS Mean ± SE**		**LS Mean ± SE**		**LS Mean ± SE**			**LS Mean ± SE**		**LS Mean ± SE**		**LS Mean ± SE**		**LS Mean ± SE**		**LS Mean ± SE**		**LS Mean ± SE**	
*Children with observed meals and intake measured ^1^*	74		54		77		65		270			75		58		84		65		282			
Meal duration (min)																							
	4.92 ± 0.41		5.18 ± 0.44		5.94 ± 0.43		6.07 ± 0.44		5.53 ± 0.29		0.0762	5.26 ± 0.38		6.59 ± 0.43		8.13 ± 0.41		8.49 ± 0.43		7.12 ± 0.27		<0.0001	<0.0001
Amount of food contained in the bowl before consumption (g)																							
	162 ± 16.1		155 ± 17.0		131 ± 17.2		184 ± 17.7		158 ± 12.0		0.0903	178 ± 15.6		168 ± 16.7		149 ± 17.6		235.2 ± 63.8		180 ± 11.7		0.0044	0.0715
<100 g	21	28	7	13	10	13	0	0	38	14		6	8	5	9	4	5	0	0	15	5		
>200 g	10	14	9	17	0	0	29	45	48	18		17	23	15	26	2	2	47	72	81	29		
Food intake per meal (g consumed)																							
	37.9 ± 6.5		41.7 ± 7.0		39.7 ± 7.0		47.0 ± 7.1		41.6 ± 4.9		0.6968	77.0 ± 6.2		81.7 ± 6.8		79.5 ± 6.7		100.9 ± 7.0		84.8 ± 4.7		0.0209	0.0831
Food intake per meal per BW (g consumed/kg of BW)																							
	5.46 ± 0.9		5.69 ± 1.0		5.44 ± 1.0		6.45 ± 1.0		5.76 ± 0.7		0.7858	10.6 ± 0.9		11.0 ± 0.9		10.8 ± 0.9		13.3 ± 1.0		11.4 ± 0.6		0.0727	0.2309
<10 g/kg BW/meal	63	85	44	81	66	86	49	75	222	82		44	59	31	53	51	61	25	39	151	54		
10–20 g/kg BW/meal	10	14	10	19	11	14	15	23	46	17		29	39	23	40	32	38	32	49	116	41		
20–30 g/kg BW/meal	1	1	0	0	0	0	0	0	1	0.5		0	0	0	0	0	0	0	0	0	0		
≥30 g/kg BW/meal	0	0	0	0	0	0	1	2	1	0.5		2	2	4	7	1	1	8	12	15	5		
Percentage of gastric capacity ^2^																							
	18.2 ± 3.0		19.0 ± 3.2		18.1 ± 3.2		21.5 ± 3.2		19.2 ± 2.3		0.7858	35.3 ± 2.8		36.7 ± 3.1		36.0 ± 3.1		44.2 ± 3.2		38.0 ± 2.1		0.0727	0.2309
Children who ate the whole portion ^3^																							
At least at one observation	6	8	2	4	2	3	0	0	10	4	NA	1	1	5	9	3	4	0	0	9	3	NA	NA
Mean energy intake per meal ^4^ (kcal/meal)																							
	14.0 ± 4.0 [6.03–22.1]		13.0 ± 4.4 [4.27–21.7]		30.7 ± 4.2 [22.1–39.2]		34.0 ± 4.3 [25.4–42.7]		22.9 ± 2.9 [16.9–28.9]		0.0001	32.6 ± 3.7 [25.1–40.2]		33.8 ± 4.2 [25.4–42.3]		60.0 ± 4.0 [51.8–68.2]		73.7 ± 4.2 [65.1–82.2]		50.0 ± 2.7 [44.3–55.7]		<0.0001	<0.0001
Contribution of two meals to the energy requirements ^4^ (% EAR from complementary foods)																							
	15.5 ± 3.1		14.6 ± 3.4		31.4 ± 3.3		36.2 ± 3.3		24.4 ± 2.2		<0.0001	21.9 ± 2.9		22.8 ± 3.3		40.1 ± 3.1		49.3 ± 3.3		33.5 ± 2.1		<0.0001	0.2497
Breastfed in the previous hour																							
At one of the two observations	28	38	27	50	38	49	33	51	126	47	0.5015	18	24	18	31	10	12	10	15	56	20	0.0333	0.0007
At both observations	35	47	19	35	33	43	26	40	113	42		46	61	40	69	65	77	49	75	200	71		
Ate in the previous hour																							
At one observation	29	39	21	39	17	22	16	25	83	31	0.3412	15	20	13	22	19	23	8	12	55	20	0.0033	0.0009
At both observations	5	7	2	4	2	3	0	0	9	3		16	21	18	31	20	24	4	6	58	21		
Child usual appetite ^5^																							
Small												14	19	9	16	1	1	8	12	32	11	NA	NA
Normal												61	81	45	77	73	87	47	72	226	80		
Large												0	3	4	7	10	12	10	16	24	9		
Child appetite on the observation day compared with usual																							
Less than usual	15	20	15	28	14	18	10	15	54	20	0.1876	15	20	19	33	5	6	8	12	47	17	0.3494	
As usual	44	60	34	63	40	52	35	54	153	57		47	63	21	36	58	69	35	54	161	57		0.9386
More than usual	15	20	5	9	23	30	20	31	63	23		13	17	18	31	21	25	22	34	74	26		
Reason for meal termination																							
Infant refuses to eat more	72	97	54	100	76	99	65	100	267	99	NA	74	99	55	95	81	96	65	100	275	98	NA	NA

RF = responsive feeding, FIF = fortified infant flour, NITR = nutritionally improved traditional recipes, LS Mean = least square mean, SE = standard error, BW = body weight, EAR = estimated average requirement, ^1^ meal observations with food intake < 10 g were excluded (10 children at T0, 1 child at T3). Final analyses included 270 children at T0 (218 with two visits, 52 with one) and 282 at T3 (257 with two visits, 25 with one), ^2^ food intake/gastric capacity calculated on the basis of 30 g/kg body weight/meal, expressed as percentages, ^3^ number of observations with <10 g left in the bowl (obtained by subtracting the food intake to the food amount contained in the bowl before consumption), ^4^ based on traditional dumplings and porridges (>90% of meals observed); other dishes not estimated, [lower confidence limit-upper confidence limit] lower and upper bounds of the 95% confidence interval for the LS mean. ^5^ Question not asked at T0 because the infant had just started diversification, ^a^ *p*-value for the intervention group term in models explained by intervention group, study timepoint, socioeconomic status tercile, and infants’ age and sex as fixed effects, village and visit as random effects, ^b^ *p*-value for the group × time interaction term in models explained by study timepoint, intervention group, their interaction (group × timepoint), economic status terciles, and infants’ age and sex as fixed effects, and village and visit as random effects.

At both timepoints, the portions served were similar in the control and RF groups, smaller in the RF + FIF group and larger in the RF + NITR group, although the difference was significant only at T3. The mean portions in the RF + FIF group almost never exceeded 200 g and were very close to the portion that should be obtained by strictly following the recommendations on the packaging (140 g). The RF + NITR group had the highest number of portions > 200 g (45% at T0 and 72% at T3).

The mean food intakes were similar across groups at T0 (~40 g/meal). They were strongly increased in all groups at T3, particularly in the RF + NITR group (101 g vs. ~80 g in the other groups), resulting in a significant difference between groups at T3 (*p* = 0.0209). However, food intake remained very low: 82% of infants had food intakes < 10 g/kg BW/meal at T0. At T3, 54% of infants had intakes < 10 g/kg BW/meal and 41% between 10 and 20 g/kg BW/meal. On average, children used only 19% of their expected gastric capacity (30 g/kg BW/meal) [17] at T0 and 38% at T3.

At T0, the energy intake was significantly higher in the RF + NITR (34 kcal/meal) and RF + FIF (31 kcal/meal) groups compared with the control and RF groups (~14 kcal/meal for both) (*p* <0.001). Similarly, at T3, energy intakes were higher in the RF + FIF and RF + NITR groups (*p* < 0.001) than in the control and RF groups (~33 kcal/meal). The RF intervention alone did not increase energy intake, probably due to the consumption of low energy-dense foods. Moreover, energy intake at T3 was significantly different between the RF + FIF and RF + NITR group (60 kcal/meal vs. 74 kcal/meal; *p* = 0.0491).

By doubling these values to simulate the energy intake of two meals per day, the contribution to the infants’ energy requirements from complementary foods (200 and 300 kcal/day for 6–8-month-old (T0) and 9–11-month-old (T3) infants, respectively) was ~20–25% in the control and RF groups and 40–50% in the RF + FIF and RF + NITR groups. In all groups, <4% of infants (*n* = 10 at T0 and *n* = 9 at T3) finished the entire portion. Therefore, the portion size was not an obstacle to higher consumption.

Regarding meal patterns, despite our recommendations, ~90% (T0) and 91% (T3) of infants in all groups had been breastfed within 1 h before the food intake measurement during at least one visit, and 34% (T0) and 41% (T3) had eaten something. At T3, fewer infants in the RF + NITR group had eaten in the hour before observation compared with the other groups.

Regardless of the group or timepoint, in 98% of cases, the meal end was determined by the child’s refusal to eat more. Mothers estimated that the consumption level was similar to the usual child’s appetite. At T3, despite the observed low food intakes, four out of five mothers considered their child’s usual appetite to be “normal”.

### 3.4. Characteristics of the Dishes Consumed (Table 3)

Millet porridge and millet dumpling with curdled milk accounted for ~90% of all traditional dishes consumed by infants. Their dry matter content (DMC) was low (9.6 g DM/100 g for porridge and 11.6 g DM/100 g for dumpling) as well as their energy densities (35.0 kcal/100 g and 42.9 kcal/100 g, respectively).

**Table 3:** Dry matter content and energy density of the dishes consumed and the corresponding intakes at T0 and T3.

					T0			T3		
		Number of FG in the Dish	Dry Matter Content Mean ± SD (g DM/100 g)	Mean Energy Density (kcal/100 g)	N obs	Food Intake (g) (Mean ± SD)	Energy Intake (kcal/meal) (Mean ± SD)	N obs	Food Intake (g) (Mean ± SD)	Energy Intake (kcal/meal) (Mean ± SD)
TF	Traditional millet porridge	1	9.6 ± 2.9	35.0	133	45.0 ± 37.9	15.8 ± 13.3	142	77.9 ± 49.9	27.3 ± 17.5
	Millet dumpling with curdled milk	2	11.6 ± 6.9	42.9	62	40.4 ± 33.8	17.3 ± 14.5	89	61.9 ± 37.3	26.5 ± 16.0
FIF	FIF Garin Yaara™ porridge	1/2 ^a^	21.3 ± 5.7	92.8	138	40.7 ± 29.9	37.9 ± 27.8	68	74.7 ± 43.9	70.2 ± 40.5
	FIF Misola™ porridge	1/2 ^a^	22.7 ± 2.6	99.0	0	-	-	95	64.7 ± 39.0	54.2 ± 31.8
NITR	Millet porridge with groundnut cake	2	17.3 ± 3.2	69.5	33	62.2 ± 42.5	38.3 ± 26.2	31	80.3 ± 51.6	49.5 ± 31.8
	White maize porridge with fish powder	2 ^b^	16.2 ± 10.0	80.4	8	44.0 ± 37.1	32.8 ± 27.7	6	98.3 ± 95.1	73.3 ± 70.9
	White maize porridge with cowpeas	2	13.8 ± 4.2	68.5	*5*	*32.5 ± 30.0*	*21.1 ± 19.5*	7	73.0 ± 41.9	47.5 ± 27.3
	Millet porridge with egg yolk	2	15.0 ± 2.2	67.1	11	68.9 ± 63.3	37.5 ± 34.4	6	139.2 ± 44.6	75.7 ± 24.3
	Millet porridge with cowpeas and moringa powder	3	12.5 ± 1.1	54.9	14	38.3 ± 19.9	19.8 ± 10.3	8	100.5 ± 52.6	52.0 ± 27.2
	Millet porridge with milk powder	2	17.3 ± 5.6	84.6	13	67.2 ± 39.7	54.3 ± 32.1	19	98.4 ± 61.0	79.4 ± 49.3
	Millet porridge with baobab pulp and groundnut cake	3	12.4 ± 3.3	49.2	16	41.4 ± 36.9	17.8 ± 15.9	18	130.3 ± 48.1	56.1 ± 20.7
	Millet dumpling with oil	2	19.6 ± 6.0	92.1	*2*	*9.5 ± 4.9*	*7.3 ± 3.8*	9	98.3 ± 44.1	75.5 ± 33.8
	Cowpea puree	2	16.0 ± 2.6	75.5	12	52.3 ± 32.3	37.2 ± 23.0	7	73.9 ± 49.3	52.6 ± 35.1
	Millet porridge with cowpea flour	2	15.1 ± 2.8	73.9	6	36.2 ± 23.1	29.1 ± 18.6	13	81.1 ± 47.3	65.3 ± 38.1

TF = traditional food, FIF = fortified infant flour, NITR = nutritionally improved traditional recipes, FG = food groups, SD = Standard deviation, N obs = number of observations. Recipes consumed ≤5 times are in italics, ^a^ 2 food groups if pulses, nuts and seeds are included, ^b^ 2 food groups because fish powder is present in significant quantities.

Due to amylase addition [34], FIF porridges presented the highest DMC (21.3 g DM/100 g for Garin Yaara™ and 22.7 g DM/100 g for Misola™) and consequently, the highest energy densities (92.8 kcal/100 g for Garin Yaara™ and 99.0 kcal/100 g for Misola™), close to the target values associated with their recommended preparation.

The DMC of NITR porridges varied widely across recipes, from 12.4 g DM/100 g to 19.6 g DM/100 g. However, it remained below the targeted DMC initially established during recipe development. Energy density also differed substantially across NITRs, from 49.2 kcal/100 g to 92.1 kcal/100 g.

Although FIF porridges had the highest energy densities, they were consumed in smaller amounts than NITR porridges, which resulted in substantially higher total energy intake for NITR dishes. Conversely, energy density and energy intake of traditional dishes were low across timepoints. Traditional porridge provided only 15–28 kcal per meal, based on mean intakes of 40 to 78 g. Energy intakes were higher for FIF and NITR and increased from T0 to T3: from 37.9 to 70.2 kcal per meal for Garin Yaara™ porridge, from 32.8 to 73.3 kcal for fish powder porridge, and from 54.3 to 79.4 kcal for milk powder porridge.

At T0, the NITR porridges with the highest mean food intakes were those enriched with egg yolk (68.9 g), powdered milk (67.2 g) and groundnut cake (62.2 g), whereas porridges with cowpea were the least consumed (mean intake < 40 g). At T3, intakes increased significantly for all recipes (on average, +95% compared with T0).

### 3.5. Contribution to Nutritional Requirements

The contribution of all dishes to the infant’s nutritional requirements was calculated based on their nutritional values and their mean intakes during a meal (Table 4). The contribution of traditional millet-based porridge and dumplings with curdled milk was small, rarely exceeding 20% of the recommended intake for energy, protein, or micronutrients. These dishes lacked vitamin A, B12 and C because they are mainly made of cereals. Their iron and zinc contents were also low, but they provided ~40% of the recommended daily intake of folate.

**Table 4:** Contribution of a meal to the infants’ estimated average requirements (EAR), taking into account individual food intakes.

					Energy	Protein	Fat	Magnesium	Calcium	Iron	Zinc
EAR/RNI per Day for 6–8-Month-Old Infants					200 kcal	2.2 g	10.5 g	43.3/51.9 mg	179.2/215 mg	7.8/9.3 mg	2.6/3.3 mg
EAR/RNI per Day for 9–11-Month-Old Infants					300 kcal	3.1 g	16.2 g	44.5/53.4 mg	190.0/228 mg	7.8/9.3 mg	2.7/3.4 mg
				*n*	% of EAR (mean ± SD) [min–max]						
T0	TF	Traditional millet porridge		133	9 ± 6 (2–41)	21 ± 15 [4–97]	3 ± 2 [1–14]	11 ± 8 [2–49]	1 ± 0 [0–3]	3 ± 2 [1–13]	5 ± 4 [1–23]
		Millet dumpling with curdled milk		62	11 ± 8 [3–35]	24 ± 16 [6–74]	4 ± 2 [1–11]	12 ± 8 [3–38]	1 ± 1 [0–4]	3 ± 2 [1–10]	6 ± 4 [1–17]
	FIF	FIF Garin Yaara™ porridge		138	17 ± 11 [4–56]	76 ± 49 [17–251]	9 ± 6 [2–31]	56 ± 36 [12–186]	18 ± 12 [4–59]	25 ± 16 [6–82]	22 ± 14 [5–73]
		FIF Misola™ porridge		0	-	-	-	-	-	-	-
	NITR	Millet porridge with groundnut cake		33	22 ± 14 [3–52]	98 ± 62 [15–236]	7 ± 5 [1–18]	33 ± 21 [5–80]	2 ± 1 [0–5]	6 ± 4 [1–14]	15 ± 9 [2–35]
		White maize porridge with fish powder		8	18 ± 12 [4–35]	75 ± 51 [16–142]	21 ± 14 [4–40]	16 ± 11 [3–30]	19 ± 13 [4–36]	5 ± 3 [1–9]	10 ± 7 [2–20]
		White maize porridge with cowpea		*5*	*15 ± 12 [6–32]*	*27 ± 22 [10–58]*	*12 ± 10 [4–25]*	*15 ± 12 [5–31]*	*1 ± 1 [0–2]*	*2 ± 2 [1–5]*	*4 ± 3 [2–9]*
		Millet porridge with egg yolk		11	27 ± 21 [6–80]	66 ± 51 [16–198]	16 ± 12 [4–48]	19 ± 14 [4–56]	2 ± 2 [1–7]	5 ± 4 [1–16]	10 ± 8 [2–31]
		Millet porridge with cowpea and moringa powder		14	16 ± 6 [6–25]	33 ± 12 [13–53]	9 ± 3 [3–14]	20 ± 7 [8–31]	10 ± 4 [4–15]	5 ± 2 [2–8]	4 ± 2 [2–7]
		Millet porridge with milk powder		13	33 ± 20 [8–70]	64 ± 38 [16–135]	24 ± 14 [6–51]	19 ± 11 [5–39]	19 ± 11 [5–40]	4 ± 2 [1–7]	10 ± 6 [3–21]
		Millet porridge with baobab pulp and groundnut cake		16	19 ± 12 [5–48]	62 ± 42 [15–162]	5 ± 3 [1–12]	24 ± 16 [6–63]	4 ± 2 [1–9]	5 ± 3 [1–12]	10 ± 7 [2–25]
		Millet dumpling with oil		*2*	*4*	*15*	*4*	*5*	*4*	*1*	*2*
		Cowpea puree		12	19 ± 10 [3–41]	76 ± 42 [14–166]	19 ± 10 [3–41]	43 ± 23 [8–93]	4 ± 2 [1–10]	7 ± 4 [1–14]	11 ± 6 [2–23]
		Millet porridge with cowpea flour		6	14 ± 8 [4–23]	30 ± 18 [9–51]	14 ± 8 [4–23]	16 ± 10 [5–27]	1 ± 1 [0–2]	3 ± 2 [1–5]	5 ± 3 [2–9]
T3	TF	Traditional millet porridge		142	10 ± 5 [1–30]	24 ± 14 [3–75]	3 ± 2 [0–10]	17 ± 10 [2–52]	1 ± 0 [0–3]	5 ± 3 [1–14]	8 ± 4 [1–24]
		Millet dumpling with curdled milk		89	10 ± 6 [2–27]	23 ± 13 [4–61]	3 ± 2 [0–8]	16 ± 9 [2–42]	2 ± 1 [0–5]	4 ± 2 [1–11]	7 ± 4 [1–19]
	FIF	FIF Garin Yaara™ porridge		68	20 ± 11 [3–45]	93 ± 51 [12–211]	11 ± 6 [1–24]	94 ± 52 [12–215]	29 ± 16 [4–66]	43 ± 23 [6–98]	37 ± 20 [5–84]
		FIF Misola™ porridge		95	17 ± 10 [3–46]	87 ± 50 [13–233]	11 ± 6 [2–29]	80 ± 46 [12–214]	28 ± 16 [4–74]	38 ± 22 [6–101]	34 ± 19 [5–89]
	NITR	Millet porridge with groundnut cake		31	19 ± 11 [3–44]	91 ± 53 [16–212]	6 ± 4 [1–15]	42 ± 24 [7–98]	3 ± 2 [0–6]	7 ± 4 [1–17]	18 ± 11 [3–43]
		White maize porridge with fish powder		6	26 ± 22 [3–62]	114 ± 94 [14–270]	29 ± 24 [4–70]	33 ± 28 [4–79]	38 ± 31 [5–90]	10 ± 9 [1–25]	21 ± 18 [3–51]
		White maize porridge with cowpea		7	19 ± 11 [5–32]	38 ± 22 [10–62]	15 ± 9 [4–25]	28 ± 16 [7–46]	2 ± 1 [1–3]	4 ± 3 [1–7]	8 ± 4 [2–13]
		Millet porridge with egg yolk		6	31 ± 10 [20–48]	81 ± 26 [52–125]	18 ± 6 [12–28]	31 ± 10 [20–48]	4 ± 1 [2–6]	9 ± 3 [6–14]	17 ± 5 [11–26]
		Millet porridge with cowpea and moringa powder		8	25 ± 13 [2–43]	55 ± 29 [5–97]	13 ± 7 [1–23]	45 ± 24 [4–79]	21 ± 11 [2–37]	12 ± 6 [1–21]	10 ± 5 [1–18]
		Millet porridge with milk powder		19	32 ± 20 [4–69]	67 ± 41 [7–142]	23 ± 14 [3–49]	27 ± 16 [3–56]	26 ± 16 [3–56]	5 ± 3 [1–11]	14 ± 9 [2–30]
		Millet porridge with baobab pulp and groundnut cake		18	31 ± 11 [11–52]	109 ± 40 [40–185]	7 ± 3 [3–12]	59 ± 22 [22–99]	8 ± 3 [3–14]	11 ± 4 [4–19]	23 ± 9 [9–39]
		Millet dumpling with oil		9	22 ± 10 [10–35]	79 ± 36 [35–126]	22 ± 10 [10–35]	35 ± 16 [15–56]	28 ± 13 [12–44]	7 ± 3 [3–11]	17 ± 8 [8–28]
		Cowpea puree		7	19 ± 10 [2–32]	81 ± 43 [11–137]	18 ± 10 [2–31]	62 ± 33 [8–105]	6 ± 3 [1–10]	10 ± 5 [1–17]	15 ± 8 [2–26]
		Millet porridge with cowpea flour		13	18 ± 11 [5–43]	42 ± 24 [12–100]	17 ± 10 [5–41]	31 ± 18 [9–74]	2 ± 1 [1–5]	6 ± 3 [2–14]	10 ± 6 [3–24]
						Vitamin A	Vitamin C	Thiamine (vitamin B1)	Folate (vitamin B9)		Cobalamin (Vitamin B12)
EAR/RNI per day for 6–8-month-old children						70 µg RAE	3.0/3.6 mg	0.17/0.2 mg	19.92/23.9 µg		0.08/0.1 µg
EAR/RNI per day for 9–11 month-old children						92 µg RAE	4.5/5.4 mg	0.20/0.2 mg	23.00/27.6 µg		0.10/0.1 µg
					*n*	% of EAR (mean ± SD) [min-max]					
T0	TF	Traditional millet porridge			133	0 ± 0	0 ± 0	6 ± 4 [1–28]	31 ± 22 [6–143]		0 ± 0
		Millet dumpling with curdled milk			62	1 ± 0 [0–2]	0 ± 0	9 ± 6 [2–26]	36 ± 24 [9–111]		0 ± 0
	FIF	FIF Garin Yaara™ porridge			138	45 ± 29 [10–149]	117 ± 76 [26–387]	18 ± 12 [4–61]	41 ± 26 [9–135]		62 ± 40 [14–205]
		FIF Misola™ porridge			0	-	-	-	-		-
	NITR	Millet porridge with groundnut cake			33	0 ± 0	0 ± 0	19 ± 12 [3–46]	66 ± 42 [10–158]		0 ± 0
		White maize porridge with fish powder			8	42 ± 28 [9–79]	2 ± 1 [0–3]	9 ± 6 [2–18]	24 ± 16 [5–46]		1166 ± 795 [242–2222]
		White maize porridge with cowpea			*5*	*24 ± 20 [9–51]*	*1 ± 1 [0–3]*	*7 ± 5 [2–14]*	*28 ± 23 [11–60]*		*0 ± 0*
		Millet porridge with egg yolk			11	21 ± 16 [5–62]	0 ± 0	14 ± 11 [3–43]	81 ± 62 [19–243]		131 ± 101 [31–393]
		Millet porridge with cowpea and moringa powder			14	18 ± 7 [7–29]	46 ± 17 [18–73]	18 ± 7 [7–28]	37 ± 14 [15–58]		0 ± 0
		Millet porridge with milk powder			13	44 ± 26 [11–93]	16 ± 9 [4–33]	12 ± 7 [3–25]	41 ± 24 [10–85]		84 ± 50 [21–176]
		Millet porridge with baobab pulp and groundnut cake			16	0 ± 0	93 ± 62 [23–242]	12 ± 8 [3–32]	42 ± 28 [10–110]		0 ± 0
		Millet dumpling with oil			*2*	*7*	*3*	*3*	*11*		*16*
		Cowpea puree			12	44 ± 24 [8–96]	65 ± 36 [12–142]	23 ± 13 [4–50]	90 ± 49 [16–196]		0 ± 0
		Millet porridge with cowpea flour			6	26 ± 16 [8–44]	0 ± 0	7 ± 4 [2–12]	40 ± 24 [11–67]		0 ± 0
T3	TF	Traditional millet porridge			142	0 ± 0	0 ± 0	8 ± 5 [1–26]	44 ± 25 [5–135]		0 ± 0
		Millet dumpling with curdled milk			89	1 ± 0 [0–2]	0 ± 0	10 ± 5 [2–26]	42 ± 23 [7–112]		0 ± 0
	FIF	FIF Garin Yaara™ porridge			68	59 ± 32 [8–135]	135 ± 74 [17–307]	27 ± 15 [4–62]	61 ± 33 [8–139]		85 ± 47 [11–195]
		FIF Misola™ porridge			95	53 ± 31 [8–142]	120 ± 70 [18–321]	23 ± 13 [4–62]	43 ± 25 [7–115]		71 ± 43 [11–194]
	NITR	Millet porridge with groundnut cake			31	0 ± 0	0 ± 0	21 ± 12 [4–50]	74 ± 43 [13–173]		0 ± 0
		White maize porridge with fish powder			6	68 ± 56 [8–161]	3 ± 2 [0–6]	17 ± 14 [2–41]	44 ± 37 [5–106]		1998 ± 1662 [246–4752]
		White maize porridge with cowpea			7	36 ± 21 [9–59]	2 ± 1 [0–3]	11 ± 6 [3–18]	48 ± 27 [12–79]		0 ± 0
		Millet porridge with egg yolk			6	27 ± 9 [18–42]	0 ± 0	21 ± 7 [14–32]	121 ± 39 [78–187]		181 ± 58 [117–280]
		Millet porridge with cowpea and moringa powder			8	33 ± 17 [3–57]	71 ± 37 [7–125]	35 ± 18 [4–62]	74 ± 39 [7–130]		0 ± 0
		Millet porridge with milk powder			19	49 ± 31 [6–105]	15 ± 9 [2–33]	15 ± 9 [2–31]	51 ± 32 [6–109]		98 ± 61 [11–209]
		Millet porridge with baobab pulp and groundnut cake			18	0 ± 0	153 ± 57 [57–259]	26 ± 10 [10–44]	91 ± 33 [33–153]		0 ± 0
		Millet dumpling with oil			9	43 ± 19 [19–68]	15 ± 7 [7–24]	20 ± 9 [9–31]	73 ± 33 [32–115]		98 ± 44 [43–156]
		Cowpea puree			7	50 ± 27 [7–85]	65 ± 35 [9–110]	29 ± 16 [4–50]	116 ± 62 [15–198]		0 ± 0
		Millet porridge with cowpea flour			13	39 ± 23 [11–92]	0 ± 0	12 ± 7 [3–29]	67 ± 39 [19–159]		0 ± 0

TF = traditional food, FIF = fortified infant flour, NITR = nutritionally improved traditional recipes, SD = standard deviation, RNI = recommended nutrient intake. Nutrient intakes per recipe were determined for each infant based on the amount consumed. Retention factors were taken into account. Then, the contribution to the estimated average requirement (EAR) was calculated for each nutrient, subtracting nutrient intakes from breast milk [17]. Iron and zinc bioavailability were estimated at 10% and 30% respectively (medium). Recipes consumed ≤5 times are presented in italics, Sources [21,27,30,31,32,35]. FIF-based porridges showed the best nutritional performance, including a very high contribution to the protein and magnesium (94% at T3), vitamin C (>100%), iron and zinc (43% at T3), vitamin A and B12, and folate requirements. Importantly, all NITRs contributed at most 12% of the RNI for iron.

Concerning NITRs, fish powder was very rich in vitamins B12 and A. For instance, the NITR maize porridge with fish powder contributed to >1000% of the vitamin B12 RNI. Egg yolk and powdered milk provided protein, folate and vitamin B12. The combination of baobab fruit pulp and groundnut cake to enrich millet porridge covered >100% of the protein and vitamin C, 59% of magnesium and >90% of folate requirements. Clearly, NITR diversity was necessary to contribute to the various micronutrient requirements, but in all cases iron intakes remained inadequate.

## 4. Discussion

The infants’ nutritional status in this Niger area is worrying. At T0, 52% of infants in our sample had anemia and 29% were stunted, far exceeding the thresholds of 40% and 20–30% respectively, considered to constitute serious public health problems [33,36]. These results are consistent with data from the latest SMART survey [2] in the same region [1].

Dietary diversity was low in the control and RF groups (traditional diet) and only 30% achieved the MDD at T0 and 55% at T3. Nevertheless, these figures are higher than those reported in the latest SMART survey [2], where only 5.9% of 6–23-month-old infants in the Zinder region achieved MDD. Low dietary diversity is associated with a higher risk of micronutrient deficiencies [37,38]. The study’s food solutions had different effects on dietary diversity. At T3, MDD was 44% in the RF + FIF group, but increased to 71% when considering the PNS group (FIF-DDS). Indeed, in the current recommendations, only the cereal, roots, tubers and plantain group is taken into account for DDS calculation when porridge from infant flours is consumed [3]. However, the Garin Yaara and Misola flour formulations include several ingredients from the PNS group, thus justifying their inclusion in the DDS calculation to better reflect the diet nutritional quality. NITRs further increased dietary diversity to such an extent that 81% of infants in the RF + NITR group achieved MDD at T3. Indeed, each NITR included at least two and sometimes three food groups (Table 3) and ingredients were provided to prepare two different NITRs per day, which enables diversity points to be accumulated.

Overall, food intakes were low. A high proportion of infants (>90%) were breastfed within one hour prior to the food intake measurement across all groups, which represents an important contextual factor when interpreting complementary food intake. Breastfeeding prior to complementary feeding has been shown to reduce intake of solid foods during meals, likely due to short-term satiety effects, and may influence appetite regulation during infancy [39,40].

In the control and RF groups, infants mainly consumed foods with low energy and nutrient density, such as traditional millet-based preparations with a very limited contribution to nutritional requirements. This situation is particularly concerning when young children frequently experience poor appetite [13]. In the RF + FIF group, food intakes were also low, probably due to the high energy density of FIF porridges. Indeed, several studies showed that energy-dense foods are associated with lower consumed quantities in under- and over-nutrition contexts, and independently of age [13,41,42]. Another explanation could be the diet monotony (children received the same FIF porridge twice per day) that may reduce appetite over time, particularly during complementary feeding when infants are developing food preferences and responsiveness to sensory stimuli [43]. Moreover, the palatability of FIF porridges was not scored very high by mothers (Supplementary Table S6a).

Conversely, NITR dishes were consumed in various quantities, but all higher than traditional foods and FIF porridges. They were also associated with longer meal durations. A hypothesis is that the observed higher intakes may be related to the greater sensory diversity of NITRs, which differ in terms of colors, textures, tastes, and flavors [43]. They were also widely appreciated by the mothers (Supplementary Table S6b). Beyond these observations, sensory diversity has been suggested in the literature to influence children’s responsiveness to food and feeding interactions. Early exposure to a variety of sensory food properties has been associated with the development of food preferences and eating behavior, and may contribute to child development through increased sensory stimulation during mealtimes [44].

Lastly, at both timepoints, energy intakes were significantly higher in the groups receiving food solutions than in the groups consuming a traditional diet. This, despite the low food intakes observed in the RF + FIF group. Despite this low dietary intake, the overall energy balance remains positive. It has in fact been reported that high-energy-dense porridges could reduce food intake whilst still allowing for higher energy (and nutritional) intake [33]. Noticeably, energy intakes were even higher in the RF + NITR group.

Poor appetite is common in contexts with high prevalence of undernutrition [16]. The observed food intake was lower than the gastric capacity of 20–30 g food/meal expected for that age [17]. This may limit the impact of nutritional strategies if not addressed. Therefore, increasing food intake is a key objective because higher intakes of nutritionally adequate foods would directly improve the food contributions to the nutritional requirements. Although the RF intervention increased meal duration, it did not significantly increase food intake compared with the control group. This suggests that while caregivers may have adopted some of the recommended practices—as reflected in longer mealtimes—the intensity of the RF intervention, as implemented, may not have been sufficient to induce behavioral changes large enough to increase children’s food intakes. More intensive approaches, such as closer follow-up, repeated counseling sessions, peer-to-peer support, practical demonstrations, home visits and personalized guidance, could further enhance caregivers’ practices and effectively improve food intake. This also raises questions about the reversibility of a poor appetite, which could result from physiological factors such as chronic intestinal inflammation.

The analysis of nutrient intakes from NITRs showed that no single recipe could meet all nutritional requirements of young children, underlining the importance of diversification. But according to the recipe, they provided appreciable amounts of essential nutrients, including lipids and folate (vitamin B9), and offered good intakes of magnesium, vitamin A and thiamine. However, none of the non-fortified recipes provided adequate iron intake. This is consistent with existing evidence showing that without fortification, it is extremely difficult to meet the young children’s iron requirements, especially in this Sahelian region, where iron-rich foods such as animal-source foods are not readily accessible and where iron deficiency is estimated to explain ~25% of anemia cases [45,46]. Animal-source foods are considered the most efficient dietary sources of bioavailable iron, and their inclusion in complementary feeding has been consistently associated with improved iron status in young children thanks to higher absorption of heme iron compared with non-heme iron sources [47,48]. In many settings, improvements in dietary intake do not consistently translate into reductions in anemia in short-term interventions, highlighting the complexity of addressing micronutrient deficiencies through food-based approaches alone [49].

In the RF + FIF group, FIF, enriched with micronutrients, contributed substantially to the requirements for iron and also for zinc, vitamin A and vitamin C, particularly at T3. Nevertheless, the prevalence of anemia increased in all groups as it is often observed in such contexts during late infancy, a period characterized by rapid growth, rising iron requirements, and the progressive depletion of iron stores acquired at birth [50]. The increase was particularly high in the RF + NITR group, which could be due to lower iron bioavailability. Finally, the increase in energy and nutrient intake observed in the RF + FIF and RF + NITR groups did not result, after three months of intervention, in any improvement in the nutritional status of the children in these two groups compared to the control and RF groups. This indicates once again that while improving the adequacy of nutritional intake is necessary, it is often insufficient to restore optimal growth or adequate hemoglobin levels. One possible explanation for this lack of improvement may be repeated exposure to pathogens, leading to chronic intestinal inflammation and environmental enteric dysfunction (EED), often associated with altered gut microbiota. EED may reduce intestinal permeability and nutrient uptake, thereby limiting the effectiveness of nutritional interventions.

Several limitations should be considered when interpreting these results. First, the intervention period was limited to 3 months, which may have reduced the observable effects of the strategies, particularly on the children’s nutritional status. Second, based on the 24 h recalls, children had on average four food occasions per day (excluding breastfeeding), whereas only one meal per day was directly observed and measured. Consequently, the contribution of the observed meals to the total daily intake may not fully reflect children’s overall dietary consumption. Third, nutrient intake calculations for children consuming a traditional diet may have slightly underestimated the actual intakes because they were based solely on millet porridge and millet dumplings with curdled milk, and other potentially more nutritious dishes consumed during the day were not included in the analysis. In addition, nutrient intake estimates were based on mean recipe compositions derived from observed household preparations rather than individual recipe analyses. While this approach reduces the influence of extreme values and better reflects typical preparation practices, it may introduce some measurement error due to variability in ingredient quantities and preparation methods across households. This could have led to some imprecision in the estimation of absolute nutrient intakes and adequacy levels. Finally, baseline socioeconomic status differed between groups, with the control group being relatively less affluent than the NITR group which could partly explain lower food intakes. However, socioeconomic status was included as a covariate in multivariate models, and the association between the intervention and food intake remained significant at T3, suggesting an independent effect of the intervention. But residual confounding cannot be fully excluded, as socioeconomic status is a complex and multidimensional construct.

## 5. Conclusions

Overall, our results highlight the complementarity of the two dietary approaches studied. FIF porridges provide high micronutrient density and are well-suited for preventing micronutrient deficiencies, particularly iron deficiency. NITR offers greater dietary diversity and is well accepted by infants, especially in terms of quantities consumed. Therefore, their combination appears most appropriate for optimizing the nutritional intake of young children.

An infant and young child feeding strategy based on alternating or combined daily consumption of one serving of FIF and one or two servings of NITR per day could be considered in order to maximize the specific benefits of each strategy. For better efficiency, this would require the reformulation of the FIF mineral and vitamin premix to follow the one serving per day specifications [51]. Implementation of the NITR strategy would imply the provision of nutritious foods through food vouchers. More studies are therefore needed to confirm these findings, assess the longer-term impacts, and evaluate the feasibility and effectiveness of such combined approaches in similar contexts.

## Figures and Tables

**Table 1:** Main characteristics of participants at the study start (T0).

	Control		RF		RF + FIF		RF + NITR		Total		*p*
	*n*	%	*n*	%	*n*	%	*n*	%	*n*	%	
	Mean ± SD		Mean ± SD		Mean ± SD		Mean ± SD		Mean ± SD		
*Mother’s characteristics*	79		68		89		76		312		
Age (years)	26.5 ± 6.7		25.1 ± 6.1		25.0 ± 6.2		24.6 ± 6.1		25.2 ± 6.3		0.4618 ^a^
Number of living children under 18 years											
	4 ± 2		4 ± 2		4 ± 2		3 ± 2		4 ± 2		0.1602 ^a^
Education level											
None	51	65	46	68	56	63	54	71	207	66	0.8872 ^a^
Literate	12	15	2	3	4	5	2	3	20	7	
Primary	10	13	11	16	10	11	9	12	40	13	
Secondary (middle/high school)	6	7	9	13	19	21	11	14	45	14	
Underweight (BMI < 18.5) ^1^	13	22	10	23	18	23	17	25	58	23	0.9729 ^a^
Overweight and obesity (BMI > 25) ^1^	2	3	1	2	7	9	3	4	13	5	NA
Socioeconomic status terciles ^2^											
Poorest	39	49	22	32	21	24	22	29	104	33.3	<0.001 ^b^
Median	29	37	19	28	35	39	20	27	103	33.3	
Most affluent	11	14	27	40	33	37	33	44	104	33.3	
*Infant’s characteristics*	79		62		84		74		299		
Sex											
Girl	33	42	29	47	43	51	27	36	132	44	0.2864 ^a^
Boy	46	58	33	53	41	49	47	64	167	56	
Age (months)	7.3 ± 0.7		7.3 ± 0.7		7.3 ± 0.7		7.6 ± 0.7		7.4 ± 0.7		0.4307 ^a^
Weight (kg)	7.0 ± 1.1		7.0 ± 0.9		6.9 ± 0.9		7.3 ± 0.8		7.0 ± 0.9		0.1822 ^c^
Height (cm)	65.5 ± 3.4		65.5 ± 3.2		65.5 ± 3.1		66.7 ± 3.2		65.8 ± 3.2		0.3705 ^c^
Hemoglobin level (g/dL)	10.1 ± 1.2		10.2 ± 1.1		10.4 ± 1.2		10.1 ± 1.2		10.2 ± 1.2		0.8463 ^c^
Stunting	28	35	17	27	25	29	16	22	86	29	0.2170 ^c^
Moderate wasting ^3^	11	14	8	13	11	13	8	11	38	13	0.9066 ^c^
Anemia	43	54	35	56	39	46	41	55	158	52	0.5322 ^c^

RF = responsive feeding, FIF = fortified infant flour, NITR = nutritionally improved traditional recipes, SD = standard deviation, ^1^ control *n* = 60; RF *n* = 43; RF + FIF *n* = 78; RF + NITR *n* = 67, ^2^ one child removed from the RF + NITR group because outlier, ^3^ no severe acute malnutrition at T0 because this was an exclusion criteria, ^a^ *p*-value for the intervention group in models explained by intervention group as fixed effect and village as random effect, ^b^ *p*-value for the intervention group obtained with the chi-square test, ^c^ *p*-value for the intervention group in models explained by intervention group, socioeconomic status terciles, children’s age and sex as fixed effects, and village as random effect, Anemia cut-off: hemoglobin concentration < 10.5 g/dL [33].

## Data Availability

The data described in the manuscript and the codebook are available from the corresponding author upon reasonable request.

## Supplementary Materials

The following supporting information can be downloaded at: https://www.mdpi.com/article/10.3390/nu18132117/s1, Supplementary Table S1. Five key hygiene messages in the WASH programme promotion activities. Supplementary Table S2. Five key messages used for responsive feeding promotion. Supplementary Table S3a. Proportions of ingredients in the fortified infant flours used in the study. Supplementary Table S3b. Nutritional value of the fortified infant flours used in the study. Supplementary Figure S1. Example of a recipe sheet provided to caregivers in the RF+NITR group. Supplementary Table S4a. Housing conditions of households in the sample. Supplementary Table S4b. Housing possessions of the households in the sample. Supplementary Table S5. Anthropometry and nutritional status at T0 and T3. Supplementary Table S6a. Mothers’ assessments of the NITRs. Supplementary Table S6b. Mothers’ assessments of the FIFs.
