# Supplementary material for: Nutritionally Improved Traditional Recipes and Fortified Infant Flours to Increase the Nutritional and Energy Intake in 6–11-Month-Old Infants in Rural Niger: A Randomized Controlled Trial
Source: Nutrients. 2026 Jun 29;18(13):2117. doi: 10.3390/nu18132117 (PMC13363320; doi:10.3390/nu18132117)
Supplement: Supplementary file 1 [file nutrients-18-02117-s001.zip › nutrients-4354942-supplementary.pdf]

Supplementary Table S1. Five key hygiene messages in the WASH programme promotion activities.

**Message 1**	Wash your hands with soap after using the toilet or after bathing your child, and before preparing your child's meal.
**Message 2**	Use latrines where available.
**Message 3**	Use potable water (freshly collected) for feeding the child, ensuring that the water remains potable from the point of collection until consumption or use.
**Message 4**	Have the child sleep under an insecticide-treated mosquito net to protect them from malaria.
**Message 5**	Position the child on a mat during meals.

Supplementary Table S2. Five key messages used for responsive feeding promotion.

	At T0<br>6-8 months	At T3<br>9-11 months
**Message 1**<br>To improve a child's appetite, encourage self-feeding.	Let the child touch and explore the food and offer a second spoon to learn how to use it.	Offer small pieces of food that the child can pick up with their hands and encourage the child to feed themselves with a spoon.
**Message 2**<br>To improve contact between caregiver and child, adopt appropriate behaviour.	Face the child so that they can see your face, eyes and smile.	Give the child a toy and talk to them while keeping their attention on mealtime.
**Message 3**<br>To improve a child's appetite, do not force them to eat.	Offer water when the child refuses food, then go back to food. If the child continues to refuse, after 3 attempts, wait a moment and try again.	When the child refuses to eat, ask why and take a break. If the child continues to refuse after 3 times, try again a little later.
**Message 4**<br>To meet the child's needs and improve their appetite, provide age-appropriate quantities.	Start by giving 2-3 spoons of food and then around ½ bowl* of porridge per meal twice per day.	Give ½ bowl of porridge per meal 3 times per day or ¾ bowl of porridge 2 times a day.
**Message 5**<br>To make mealtimes enjoyable, feed your child patiently while encouraging them.	Take the time needed to feed the child, while maintaining a regular rhythm, and use positive words to praise (e.g. well done, very good), encourage (e.g. keep it up), and talk about food ("It's porridge, it's very good").	

*volume of the bowl 250 ml

Example of an illustration: "Face the child so that they can see your face, eyes and smile".

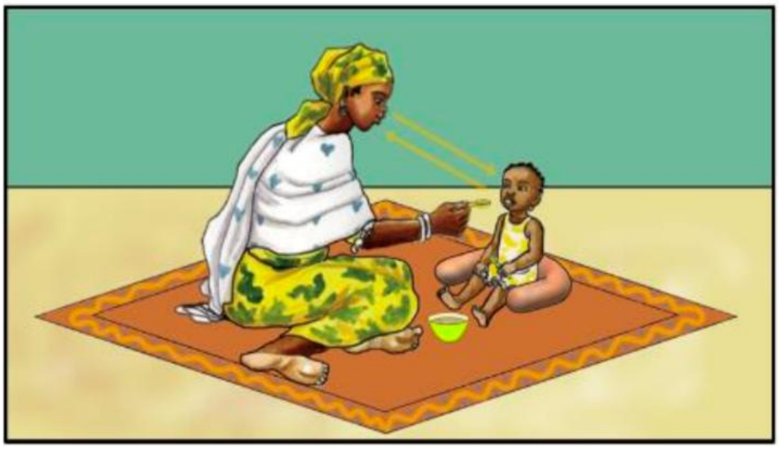

<u>Supplementary Table S3a.</u> Proportions of ingredients in the fortified infant flours used in the study

	Ingredients	*%*
Misola	Millet (whole grains)	58.43
	Soybeans (roasted hulled)	18.00
	Peanut (roasted seeds)	9.00
	Sugar (powdered)	13.00
	Salt	0.73
	$CaCO_3$	0.65
	CMV IS 2267	0.18
	Enzyme BAN	0.01
Garin Yaara	Millet (whole grains)	63.51
	Soybeans (roasted hulled)	12.00
	Peanut (roasted seeds)	8.00
	Cowpea (dry seeds)	6.00
	Sugar (powdered)	9.00
	Salt	0.69
	$CaCO_3$	0.61
	CMV IS 2267	0.18
	Enzyme BAN	0.01

Supplementary Table S3b. Nutritional value of the fortified infant flours used in the study

	Misola	***Garin Yaara***
Protein (g)	18.08	17.40
Fat (g)	11.84	10.26
Linolenic acid (mg)	404.65	355.67
Linoleic acid (mg)	5 518.42	4 806.50
Digestible protein (g)	16.10	15.28
Histidine (mg)	377.70	388.48
Isoleucine (mg)	714.54	685.90
Leucine (mg)	1 301.17	1 240.92
Lysine (mg)	695.34	650.84
Methionine + cysteine (mg)	573.09	530.80
Phenylalanine + tyrosine (mg)	1 373.11	1 260.33
Threonine (mg)	604.03	563.14
Tryptophan (mg)	228.34	220.79
Valine (mg)	802.44	784.88
Sodium (mg)	320.62	308.70
Potassium (mg)	684.01	681.89
Calcium (mg)	351.34	331.48
Chlorine (mg)	488.26	467.93
Phosphorus (mg)	348.93	354.67
Iron (mg)	19.77	20.15
Copper (µg)	638.12	656.92
Magnesium (mg)	237.54	254.56
Iodine (µg)	103.23	103.81
Zinc (mg)	6.03	6.02
Manganese (µg)	1 367.89	1 455.11
Selenium (µg)	20.41	20.12
Vitamin A (µg eq. Retinol)	344.02	343.91
Ascorbic acid (mg)	51.63	52.21
Vitamin D (µg)	9.89	10.01
Thiamine (µg)	430.83	436.33
Riboflavin (µg)	463.35	461.83
Vitamin B6 (µg)	845.89	852.28
Nicotinamide (µg)	5 926.45	5 972.30
Vitamin B12 (µg)	0.52	0.52
Folic acid (µg)	84.90	106.47
Pantothenic acid (µg)	1 091.55	1 041.99
Vitamin E (mg)	12.45	12.34
Vitamin K1 (µg)	16.28	13.78
Biotin (µg)	12.27	8.27

Supplementary Figure S1. Example of a recipe sheet provided to caregivers in the RF+NITR group

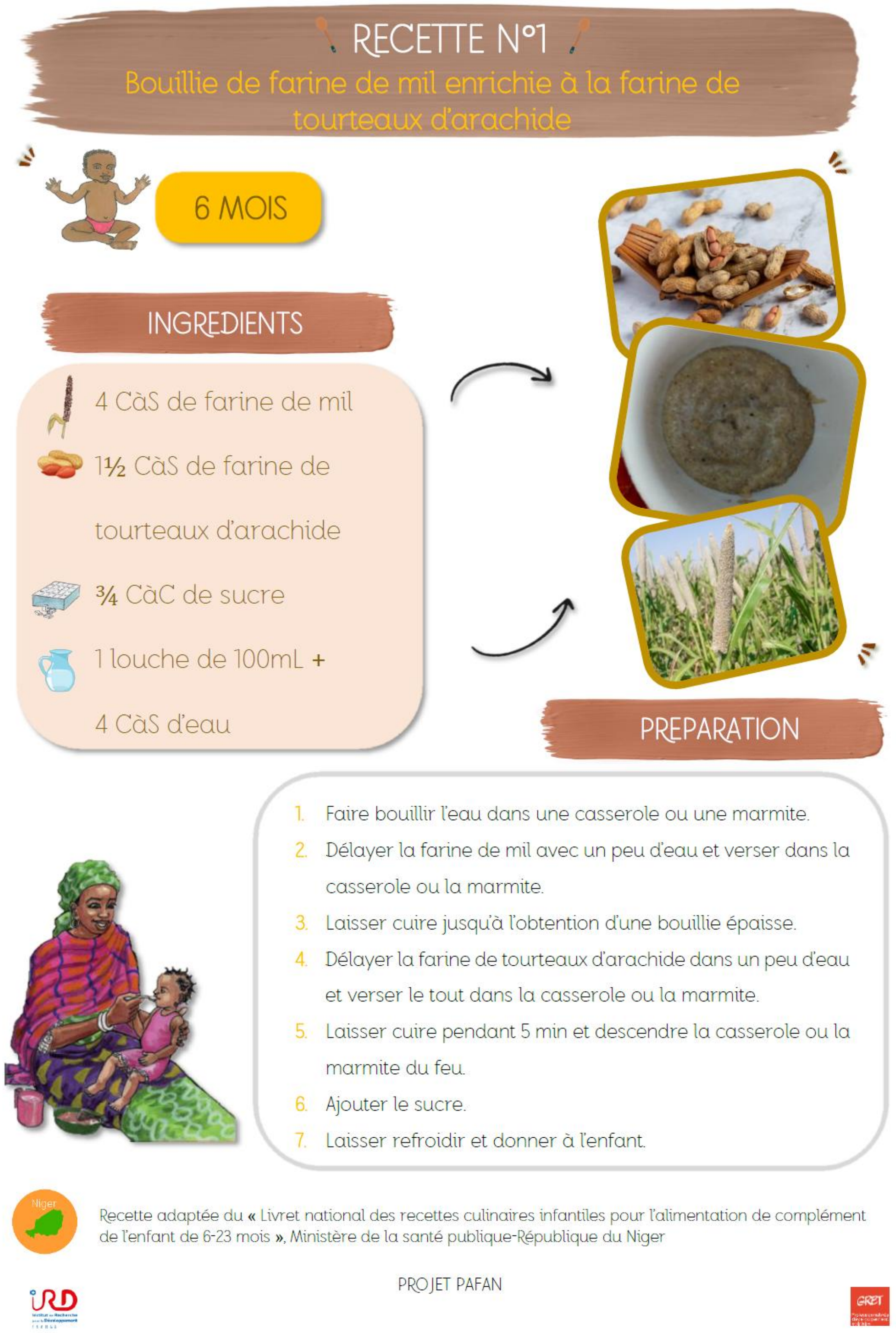

Supplementary Table S4a. Housing conditions of households in the sample

	Control		RF		RF + FIF		RF + NITR		Total	
	**n**	**%**	**n**	**%**	**n**	**%**	**n**	**%**	**n**	**%**
**Respondents**	**79**		**68**		**89**		**76**		**312**	
**Occupancy status[1]**										
Owner	79	**100**	67	**99**	88	**99**	76	**100**	310	**98**
Tenant	0	**0**	0	**0**	1	**1**	0	**0**	1	**1**
Living rent-free	0	**0**	1	**1**	0	**0**	0	**0**	1	**1**
**Number of persons in the house[2]**										
From 2 to 5	26	**33**	23	**34**	39	**44**	29	**38**	117	**37**
From 6 to 11	41	**52**	32	**47**	36	**40**	37	**49**	146	**47**
≥ 12	12	**15**	13	**19**	14	**16**	10	**13**	49	**16**
**Number of habitable rooms[2]**										
From 1 to 4	67	**85**	59	**87**	83	**93**	64	**84**	273	**88**
≥ 5	12	**15**	9	**13**	6	**7**	12	**16**	39	**12**
**Number of individuals per room[2]**										
≤ 3	35	**44**	31	**46**	47	**53**	39	**51**	152	**49**
3 ≤ 4	19	**24**	18	**26**	18	**20**	20	**26**	75	**24**
> 4	25	**32**	19	**28**	24	**27**	17	**23**	85	**27**
**Wall type**										
Cement	0	**0**	1	**2**	1	**1**	1	**1**	3	**1**
Clay (banco) / brick	52	**66**	47	**69**	78	**88**	57	**75**	234	**75**
Branch / bamboo / straw / straw hut / secco	27	**34**	20	**29**	10	**11**	18	**24**	75	**24**
**Roof type**										
Raw earth / clay	51	**65**	40	**59**	69	**78**	48	**63**	208	**67**
Sheet metal	3	**4**	12	**18**	4	**4**	7	**9**	26	**8**
Straw / secco	25	**31**	16	**23**	16	**18**	21	**28**	78	**25**
**Floor covering**										
Cement / plastic	0	**0**	7	**10**	7	**8**	5	**7**	19	**6**
Mat	30	**38**	26	**38**	29	**32**	26	**34**	111	**36**
Uncoated / earth / clay	49	**62**	35	**52**	53	**60**	45	**59**	182	**58**
**Lighting source**										
Electricity / solar panel	1	**1**	5	**7**	11	**12**	12	**16**	29	**9**
Petroleum lamp / torch (battery)	78	**99**	63	**93**	78	**88**	64	**84**	283	**91**
**Cooking fuel**										
Gas / electricity / coal	1	**1**	1	**1**	4	**4**	0	**0**	6	**2**
Wood	78	**99**	67	**99**	85	**96**	76	**100**	306	**98**
**Water source for washing and/or food**										
Home tap	0	**0**	4	**6**	11	**13**	2	**2**	17	**6**
Public tap / drinking fountain	7	**9**	30	**44**	36	**40**	25	**33**	98	**31**

Closed well (borehole)	20	**25**	15	**22**	15	**17**	18	**24**	68	**22**
Open well	52	**66**	19	**28**	27	**30**	31	**41**	129	**41**
**Latrine type**										
Individual latrines	12	**15**	15	**22**	16	**18**	18	**24**	61	**20**
Communal latrines	17	**22**	4	**6**	13	**15**	13	**17**	47	**15**
No latrines	50	**63**	49	**72**	60	**67**	45	**59**	204	**65**

[1] This variable was not used in the multiple correspondence analysis.

[2] The variables "number of persons in the house" and "number of habitable rooms" were combined to create the variable "number of individuals per room". The variable "number of individuals per room" was used in the analysis.

Supplementary Table S4b. Housing possessions of the households in the sample

	**Control**		**RF**		**RF + FIF**		**RF + NITR**		**Total**	
	**n**	**%**	**n**	**%**	**n**	**%**	**n**	**%**	**n**	**%**
**Respondents**	**79**		**68**		**89**		**76**		**312**	
**Beds**										
0	1	**1**	6	**9**	6	**7**	4	**5**	17	**5**
1	50	**63**	44	**65**	62	**70**	50	**66**	206	**66**
> 1	28	**36**	18	**26**	21	**23**	22	**29**	89	**29**
**Chairs**										
0	51	**65**	41	**60**	47	**53**	43	**57**	182	**58**
1	20	**25**	14	**21**	27	**30**	16	**21**	77	**25**
> 1	8	**10**	13	**19**	15	**17**	17	**22**	53	**17**
**Radio**	8	**10**	19	**28**	14	**16**	19	**25**	60	**19**
**Television**	0	**0**	2	**3**	2	**2**	0	**0**	4	**1**
**Bicycle**	2	**3**	1	**1**	5	**6**	8	**11**	16	**5**
**Moto**	6	**8**	14	**21**	14	**16**	20	**26**	54	**17**
**Car**	0	**0**	1	**1**	2	**2**	1	**1**	4	**1**
**Charette**	30	**38**	27	**40**	45	**51**	45	**59**	147	**47**
**Mobile phone**										
0	20	**25**	18	**27**	12	**13**	12	**16**	62	**20**
1	54	**69**	43	**63**	65	**74**	50	**66**	212	**68**
> 1	5	**6**	7	**10**	12	**13**	14	**18**	38	**12**

The 17 variables used to assess the households' socioeconomic status include 8 variables relating to housing quality and 9 variables relating to possessions.

Supplementary Table S5. Anthropometry and nutritional status at T0 and T3

	T0											T3											
	**Control**		**RF**		**RF + FIF**		**RF + NITR**		**Total**		**p** group [a]	**Control**		**RF**		**RF + FIF**		**RF + NITR**		**Total**		**p** group [a]	**p** group*time [b]
	**n**	**%**	**n**	**%**	**n**	**%**	**n**	**%**	**n**	**%**		**n**	**%**	**n**	**%**	**n**	**%**	**n**	**%**	**n**	**%**		
	Mean ± SD		Mean ± SD		Mean ± SD		Mean ± SD		Mean ± SD			Mean ± SD		Mean ± SD		Mean ± SD		Mean ± SD		Mean ± SD			
	**79**		**62**		**84**		**74**		**299**			**77**		**58**		**85**		**72**		**292**			
**Weight** (kg)	**7.0 ± 1.1**		**7.0 ± 0.9**		**6.9 ± 0.9**		**7.3 ± 0.8**		**7.0 ± 0.9**		0.1822	**7.7 ± 1.0**		**7.8 ± 0.9**		**7.5 ± 1.0**		**8.0 ± 0.9**		**7.7 ± 0.9**		0.0330	0.2190
**Height** (cm)	**65.5 ± 3.4**		**65.5 ± 3.2**		**65.5 ± 3.1**		**66.7 ± 3.2**		**65.8 ± 3.2**		0.3705	**69.3 ± 3.3**		**69.3 ± 2.6**		**68.6 ± 3.1**		**70.2 ± 3.0**		**69.3 ± 3.1**		0.4186	0.2367
**Haemoglobin concentration** (g/L)	**10.1 ± 1.2**		**10.2 ± 1.1**		**10.4 ± 1.2**		**10.1 ± 1.2**		**10.2 ± 1.2**		0.8463	**9.8 ± 1.5**		**9.9 ± 1.3**		**10.2 ± 1.3**		**9.5 ± 1.3**		**9.8 ± 1.4**		0.2608	0.4219
**Stunting**	28	**35**	17	**27**	25	**29**	16	**22**	86	**29**	0.2170	32	**41**	22	**38**	43	**50**	24	**33**	121	**42**	0.2447	0.2766
**Moderate wasting**[1]	11	**14**	8	**13**	11	**13**	8	**11**	38	**13**	0.9066	8	**10**	4	**7**	13	**15**	6	**8**	31	**11**	0.2805	0.4909
**Anaemia**[2]	43	**54**	35	**56**	39	**46**	41	**55**	158	**52**	0.5322	51	**66**	39	**67**	46	**54**	56	**78**	193	**66**	**0.0246**	0.5271

[1] *There was no severe acute malnutrition (SAM) at T0 because this was an exclusion criterion. At T3, there were 2 infants with SAM in the RF+FIF group and 1 in the RF+NITR group.*

[2]*Anaemia = haemoglobin rate < 105 g/L (WHO, 2024)*

[a] *p-value for the intervention group term in models explained by intervention group, study timepoint, socioeconomic status tercile, and infants' age and sex as fixed effects, and village and visit as random effects*

[b] *p-value for the group × time interaction term in models explained by study timepoint, intervention group, their interaction (group × timepoint), socioeconomic status tercile, and infants' age and sex as fixed effects, and village and visit as random effects*

Supplementary Table S6a. Mothers' assessments of the NITRs

	RF+NITR	
	n	%
	**65**	
**Millet porridge with peanut cake**		
Appreciated by the child (yes/no)	64	99
Appreciated by the mother		
Very much	63	97
Moderately	2	3
Not appreciated	0	0
The mother doesn't know	0	0
The mother will make the recipe again (yes/no)	65	100
**White maize porridge with fish powder**		
Appreciated by the child (yes/no)	53	82
Appreciated by the mother		
Very much	50	77
Moderately	7	11
Not appreciated	7	11
The mother doesn't know	1	1
The mother will make the recipe again (yes/no)	58	89
**White maize porridge with cowpea**		
Appreciated by the child (yes/no)	63	97
Appreciated by the mother		
Very much	61	94
Moderately	3	5
Not appreciated	1	1
The mother doesn't know	0	0
The mother will make the recipe again (yes/no)	65	100
**Millet porridge with egg yolk**		
Appreciated by the child (yes/no)	61	94
Appreciated by the mother		
Very much	61	94
Moderately	3	5
Not appreciated	1	1
The mother doesn't know	0	0
The mother will make the recipe again (yes/no)	64	99
**Millet porridge with cowpea and moringa powder**		
Appreciated by the child (yes/no)	61	94
Appreciated by the mother		
Very much	60	92
Moderately	3	5
Not appreciated	2	3
The mother doesn't know	0	0
The mother will make the recipe again (yes/no)	63	97
**Millet porridge with milk powder**		
Appreciated by the child (yes/no)	63	97
Appreciated by the mother		
Very much	61	94
Moderately	4	6
Not appreciated	0	0
The mother doesn't know	0	0

The mother will make the recipe again (yes/no)	64	**99**
**Millet porridge with baobab pulp and peanut cake**		
Appreciated by the child (yes/no)	63	**97**
Appreciated by the mother		
Very much	60	**92**
Moderately	3	**5**
Not appreciated	2	**3**
The mother doesn't know	0	**0**
The mother will make the recipe again (yes/no)	64	**99**
**Millet dumpling with oil**		
Appreciated by the child (yes/no)	61	**94**
Appreciated by the mother		
Very much	61	**94**
Moderately	3	**5**
Not appreciated	0	**0**
The mother doesn't know	1	**1**
The mother will make the recipe again (yes/no)	64	**99**
**Cowpea puree**		
Appreciated by the child (yes/no)	63	**97**
Appreciated by the mother		
Very much	62	**95**
Moderately	3	**5**
Not appreciated	0	**0**
The mother doesn't know	0	**0**
The mother will make the recipe again (yes/no)	64	**99**
**Millet porridge with cowpea flour**		
Appreciated by the child (yes/no)	63	**97**
Appreciated by the mother		
Very much	64	**99**
Moderately	1	**1**
Not appreciated	0	**0**
The mother doesn't know	0	**0**
The mother will make the recipe again (yes/no)	65	**100**

Supplementary Table S6b. Mothers' assessments of the FIFs

	RF + FIF	
	**n**	%
**FIF Garin Yaara™ porridge**	**84**	
Appreciated by the child (yes/no)	82	**98**
Appreciated by the mother		
Very much	81	**96**
Moderately appreciated by the mother	3	**4**
Not appreciated by the mother	0	**0**
Mother likes flour because it feeds their children well	37	**44**
Mother likes flour because it contains healthy ingredients	31	**37**
Mother likes flour for its taste	19	**23**
**FIF Misola™ porridge**	**76**	
Appreciated by the child (yes/no)	76	**100**
Appreciated by the mother		
Very much	70	**92**
Moderately appreciated by the mother	6	**8**
Not appreciated by the mother	0	**0**
Mother likes flour because it feeds their children well	26	**34**
Mother likes flour because it contains healthy ingredients	29	**38**
Mother likes flour for its taste	23	**30**
